# Repetitive transcranial magnetic stimulation in drug‐resistant idiopathic epilepsy of dogs: A noninvasive neurostimulation technique

**DOI:** 10.1111/jvim.15919

**Published:** 2020-10-03

**Authors:** Marios Charalambous, Luc Van Ham, Bart J. G. Broeckx, Tom Roggeman, Sofie Carrette, Kristi Vonck, Roelof R. Kervel, Ine Cornelis, Sofie F. M. Bhatti

**Affiliations:** ^1^ Small Animal Department Faculty of Veterinary Medicine, Ghent University Merelbeke Belgium; ^2^ Department of Nutrition Genetics and Ethology, Faculty of Veterinary Medicine, Ghent University Merelbeke Belgium; ^3^ Department of Neurology Ghent University Hospital, 4Brain, Ghent University Ghent Belgium

**Keywords:** dog, epileptic seizures, management, nonpharmacological, refractory

## Abstract

**Background:**

Although repetitive transcranial magnetic stimulation (rTMS) has been assessed in epileptic humans, clinical trials in epileptic dogs can provide additional insight.

**Objectives:**

Evaluate the potential antiepileptic effect of rTMS in dogs.

**Animals:**

Twelve client‐owned dogs with drug‐resistant idiopathic epilepsy (IE).

**Methods:**

Single‐blinded randomized sham‐controlled clinical trial (dogs allocated to active or sham rTMS) (I) and open‐labeled uncontrolled clinical trial (dogs received active rTMS after sham rTMS) (II). Monthly seizure frequency (MSF), monthly seizure day frequency (MSDF), and number of cluster seizures (CS) were evaluated for a 3‐month pre‐TMS and post‐rTMS period and safety was assessed. The lasting effect period of rTMS was assessed in each dog treated by active stimulation using the MSF ratio (proportion of post‐TMS to pre‐rTMS MSF) and treatment was considered effective if the ratio was <1.

**Results:**

No adverse effects were reported. In trial I, MSF and MSDF decreased significantly (*P* = .04) in the active group (n = 7). In the sham group (n = 5), no significant changes were found (*P* = .84 and .29, respectively). Cluster seizures did not change significantly in either group. No significant differences were detected between the groups. In trial II, previously sham‐treated dogs (n = 5) received active rTMS and significant decreases in MSF and MSDF were noted (*P* = .03 and .008, respectively). The overall effect of rTMS lasted for 4 months; thereafter, the MSF ratio was >1.

**Conclusions and Clinical Importance:**

Repetitive transcranial magnetic stimulation may be a safe adjunctive treatment option for dogs with drug‐resistant IE, but large‐scale studies are needed to establish firm conclusions.

**Abbreviations**
AEDs: antiepileptic drugs
CMAPs: compound muscle action potentials
CRI: constant rate infusion
CS: cluster seizures
MSDF: monthly seizure day frequency
MSF: monthly seizure frequency
rTMS: repetitive transcranial magnetic stimulation

## 1 INTRODUCTION

Idiopathic epilepsy (IE) is a common neurological disorder, with an estimated prevalence of 0.5% to 0.82% in the general canine population, and up to 33% in certain families of genetically predisposed breeds.^1^, ^2^, ^3^, ^4^, ^5^, ^6^, ^7^ Drug resistance occurs in up to 30% of the dogs with IE leading to a grave prognosis and eventually euthanasia because of limited nonpharmacological treatment options.^8^ Repetitive transcranial magnetic stimulation (rTMS) has received attention the recent years as a treatment method that can have neuromodulatory effects on the brain that last longer than the duration of the neurostimulation.^9^ Although the specific antiepileptic mechanism of action still remains unclear,^10^ it might be related to the disruption of networks related to cortical hyperexcitability.^11^, ^12^ Clinical trials of low‐frequency rTMS in drug‐resistant epilepsy in humans however had conflicting outcomes with regard to the decrease in seizure frequency.^13^, ^14^, ^15^ Because dogs with spontaneous epilepsy are similar in etiology, clinical manifestation, treatment response, and drug resistance to epileptic humans,^16^, ^17^, ^18^, ^19^ a preliminary veterinary study was conducted to (a) investigate this new, noninvasive, and nonpharmacological treatment option for dogs with drug‐resistant IE and (b) provide preliminary information for future large‐scale clinical trials in dogs that could establish firm conclusions regarding its effect in IE of dogs and a potential use as a translational model for epileptic humans.

## 2 MATERIAL AND METHODS

Dogs with drug‐resistant IE without age, breed, or sex limitations were considered for enrollment in the study. The classification, definition, and diagnosis of IE were based on the recommendations of the International Veterinary Epilepsy Task Force (IVETF) consensus reports.^20^, ^21^ Drug‐resistant IE, in particular, was defined as epilepsy with <50% decrease in monthly seizure frequency (MSF) compared to baseline after treatment with at least 2 antiepileptic drugs (AEDs) despite optimal dose, serum drug concentrations, or both.^20^, ^21^

The study consisted of 2 trials: (I) a single‐blinded randomized sham‐controlled clinical trial and (II) an open‐labeled uncontrolled clinical trial. Each trial consisted of 3 phases: (a) the baseline epileptic seizure frequency phase (ie, a 3‐month pretreatment follow‐up period of epileptic seizures to determine baseline seizure frequency and number of cluster seizures [CS], defined as ≥2 epileptic seizures over a period of 24 hours^22^); (b) the treatment period (ie, daily treatment using active or sham [inactive] rTMS for 5 consecutive days); and, (c) the evaluation period (ie, a minimum 3‐month posttreatment follow‐up period of epileptic seizure frequency and adverse events related to treatment). After the initial evaluation period of 3 months, dogs treated using active rTMS were followed as long as possible until study termination. During phases (a) and (c), owners recorded epileptic seizure events in a diary.

The study was approved by the university's ethical committee (EC 2016/30). Owner consent forms were provided and signed by the owners. The overall timespan of recruitment was 12 months.

### 2.1 Trial I

At the end of phase (a), dogs were randomly assigned to the active or sham rTMS group by using sealed envelopes. Equal numbers of entries indicating either active or sham rTMS were created and placed in envelopes. The envelopes were sealed, mixed, and randomly numbered. They were opened for each included dog following a numerical sequence starting from envelope number 1. The investigators did not know the randomization order. Owners were blinded to the chosen treatment (ie, owners were not informed about which treatment their dog would receive). The dogs were hospitalized for 5 days or on consecutive afternoons and all received sedation and IV catheters while the procedure was initiated and after the owners had left the hospital. Blood samples for CBC and serum biochemistry as well as AED serum concentration assessment were collected from all dogs at that time.

**FIGURE 1:**
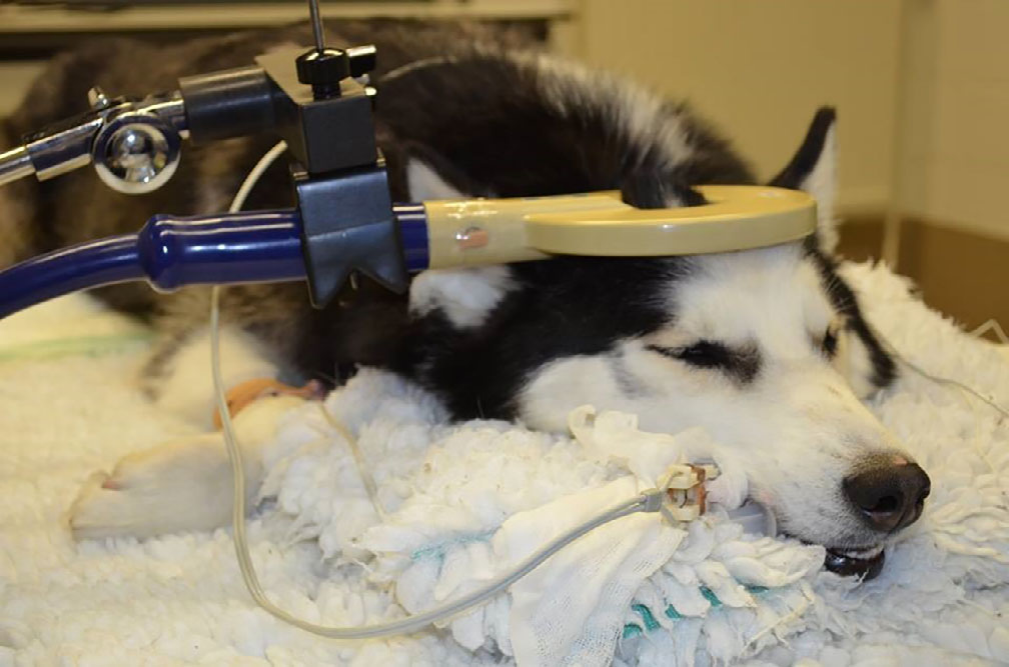
A dog in the active group under sedation during rTMS procedure. A round coil (outside diameter of 15 cm) is applied in parallel and in contact with the dog's skull with its center located at the vertex

During phase (b), the dogs received rTMS (active or sham) for 1 hour daily for 5 consecutive days. Overall, stimulation parameters and study environment were exactly the same in both groups, the only difference being that the sham group received inactive stimulation by placing the operating (round) coil perpendicular to the skull and with a distance of 20 cm above the head in order to circumvent brain stimulation. Dogs in both groups were sedated using dexmedetomidine 1 μg/kg (Dexdomitor; Orion pharma, Finland) and butorphanol 0.1 mg/kg (Dolorex; Intervet, Belgium) IV after catheter placement, and were kept under sedation during treatment using a dexmedetomidine constant rate infusion (CRI) of 1 to 3 μg/kg/h. The length of sedation was the same in both groups. Lactated Ringer's solution 5 mL kg/h (Vetivex VB11A; Dechra, UK) was administered with the dexmedetomidine CRI during treatment, and butorphanol 0.1 mg/kg was repeated 1 hour after initiation of sedation. Oxygen at 2 L/min was provided to all dogs using an anesthetic mask. Cotton earplugs were placed in the dogs' ears to avoid noise disturbances from the rTMS machine during operation. The dogs were stabilized in ventral recumbency on the examination table using tape to avoid minor movements. In dogs receiving active rTMS treatment, the round coil (outside diameter of 15 cm) was applied in parallel and in contact with the dog's skull with its center located at the vertex (active rTMS; Figure 1). Overheating of the coil was managed using cold packs and fans. The stimulation parameters were 18 trains of 90 pulses per train at a frequency of 1 Hz (ie, 1 pulse per second) and an intertrain interval of 120 seconds. Coil output was individually determined in each patient and was chosen based on the motor cortex threshold.^23^ Specifically, this threshold was defined as the minimal TMS intensity required to provoke at least 5 of 10 electromyographic responses (ie, compound muscle action potentials [CMAPs], with an amplitude of at least 50 μV, in a fully relaxed thoracic limb muscle [external carpi radialis]). The CMAPs were recorded not only to determine coil output in each patient but also to monitor stimulation of the motor cortex during rTMS treatment (active rTMS group).

During phase (c), all owners recorded epileptic seizure events in a diary and any potential adverse effects on a form. No AED dose changes were made in either group during the evaluation period. At the end of phase (c), the following variables were recorded for each dog: MSF, monthly seizure day frequency (MSDF), and monthly number of CS.^24^ The MSF before and after the treatment was calculated by counting the number of epileptic seizures per month. The MSDF before and after the treatment was calculated by counting the number of days with epileptic seizures per month. As such, the MSDF is less sensitive to bias caused by clusters with a high number of epileptic seizures. To determine the 3‐month pretreatment or posttreatment results for each dog, the MSF, MSDF, and number of CS over a period of 3 months were added and divided by 3. These values thereafter were used to calculate and compare each group's pretreatment and posttreatment MSF, MSDF, and number of CS.

### 2.2 Trial II

After the 3‐month evaluation period of trial I, dogs from the trial I sham group were included in trial II to receive active rTMS. The effect of active rTMS on these dogs was assessed for an additional 3‐month evaluation period. Owners were not blinded to the treatment in trial II. The procedure followed in each phase was the same as described earlier for the active rTMS group in trial I.

### 2.3 Statistical analysis

Statistical analysis was conducted using R version 3.5.2. Firstly, baseline values for MSF, MSDF, and number of CS of the sham (n = 5) and active rTMS (n = 7) group were compared using the Wilcoxon rank‐sum test. The Wilcoxon signed‐rank test then was used to compare the outcomes (MSF, MSDF, or CS) within each group as well as between groups. Significance was set at *P* ≤ .05. The MSF ratio (proportion of monthly post‐rTMS MSF to 3‐month pre‐rTMS MSF) was used to determine the duration of rTMS efficacy (if any) in each dog treated by active rTMS. The rTMS was considered effective if the MSF ratio remained <1.

## 3 RESULTS

### 3.1 Trial I

**FIGURE 2:**
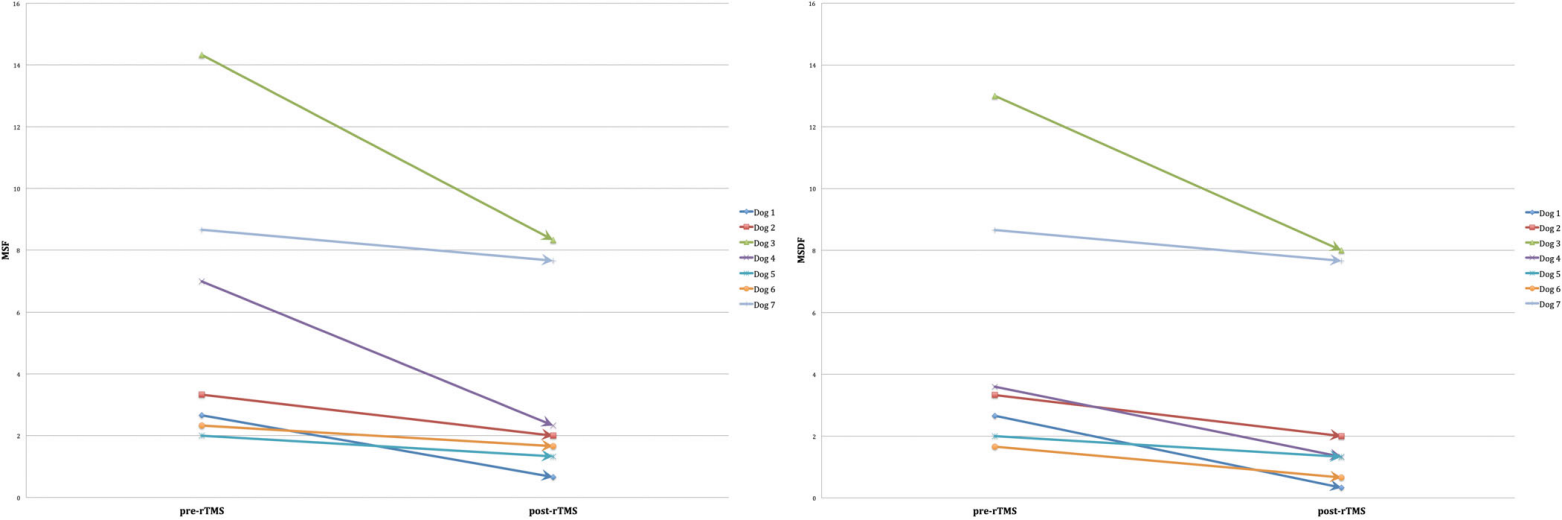
Illustration of the 3‐month pre‐rTMS and post‐rTMS MSF and MSDF for each dog in the active group

**FIGURE 3:**
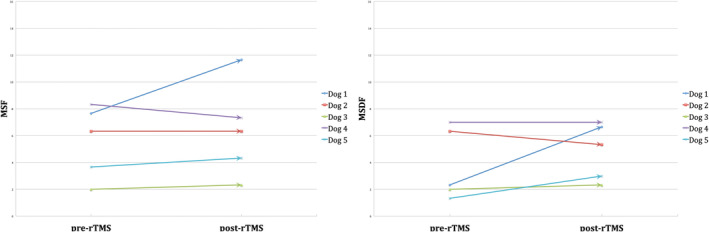
Illustration of the 3‐month pre‐rTMS and post‐rTMS MSF and MSDF for each dog in the sham group

Dogs were randomized to receive either active (n = 7) or sham (n = 5) rTMS. The difference in the number of dogs recruited between the 2 groups was a consequence of the decision of 2 owners to withdraw from the study before their dogs could be included in the trial. At baseline, no significant differences were found between the 2 groups in terms of MSF (*P* = .8), MSDF (*P* = .57), number of CS (*P* = .78), and general characteristics. Details of the baseline characteristics of all dogs in each group are provided in Table 1. It was possible to record CMAPs in all dogs stimulated by active rTMS, which successfully determined TMS coil output. Median TMS coil output was 70% (range, 70‐80%) for the active group. Eleven of 12 dogs completed the entire protocol, whereas 1 dog (in the active group) was euthanized after 2 months in the evaluation period upon the owner's request for reasons unrelated to the trial or treatment. In the active group, significant differences were observed in MSF (*P* = .046) and MSDF (*P* = .046) but not in the number of CS (*P* = .58) post‐rTMS compared to pre‐rTMS. In the sham group, no significant differences were observed in either MSF (*P* = .84), MSDF (*P* = .29), or number of CS (*P* = .12) post‐rTMS compared to pre‐rTMS. These results are summarized in Table 2 and Figures 2 and 3. When comparing the 2 groups, no significant differences were found in MSF (*P* = .14), MSDF (*P* = .25), and number of CS (*P* = .61) post‐rTMS. Based on the MSF ratio, the median rTMS effect lasted for 4 months (range, 2‐10). Median follow‐up of dogs treated with active rTMS from inclusion to termination of the study was 4 months (range, 2‐12). In the active group, no adverse effects were related to the 5‐day treatment of low‐frequency rTMS.

**TABLE 1:** Details of baseline characteristics in each group

Groups	Active rTMS	Sham rTMS
Number of dogs	7	5
Breed	Australian Shepherd, Border Collie, Cane Corso, Golden Retriever, French Bulldog, Jack Russell Terrier, Italian Spinone	Beagle, Boston Terrier, Cane Corso, Golden Retriever, American Staffordshire Terrier
Age	Median, 4.3 (range, 1.9‐6.5 years)	Median, 3.8 (range, 3.1‐9 years)
Sex/neuter status	4 intact male dogs (57%) 3 neutered female dogs (43%)	3 intact male dogs (60%) 2 neutered female dogs (40%)
Type of epileptic seizures	Generalized tonic‐clonic (7 dogs, 100%)	Generalized tonic‐clonic (5 dogs, 100%)
Tier classification	Tier I (2 dogs, 28%) and tier II (5 dogs, 72%)	Tier I (1 dog, 20%) and tier II (4 dogs, 80%)
Chronic/maintenance antiepileptic drugs	Phenobarbital/potassium bromide combination treatment (5 dogs, 70%) and phenobarbital/potassium bromide/levetiracetam combination treatment (2 dogs, 30%)	Phenobarbital/potassium bromide combination treatment (3 dogs, 60%) and phenobarbital/potassium bromide/levetiracetam combination treatment (2 dogs, 40%)
Time period on multidrug treatment	Median, 18 (range, 10‐28 months)	Median, 20 (range, 7‐25 months)
Cluster epilepsy (before occurrence of status epilepticus)	12 dogs (60%)	7 dogs (47%)

**TABLE 2:** Details of the 3‐month MSF, MSDF, and number of cluster seizures in each group pretreatment and posttreatment with a 5‐day low‐frequency rTMS

		Trial I		Trial II
		Active rTMS (n = 7)	Sham rTMS (n = 5)	Active rTMS following sham (n = 5)
MSF median (range)	Pre	3.33 (2.00‐14.33)	7.66 (2.00‐8.33)	6.33 (2.33‐11.66)
	Post	2.00 (.66‐8.33)	6.33 (2.33‐11.66)	2.66 (1.00‐8.00)
MSDF median	Pre	3.33 (1.66‐13.00)	2.33 (1.33‐7.33)	5.33 (2.33‐6.66)
(range)	Post	1.33 (.66‐8.00)	5.33 (2.33‐6.66)	2.33 (.66‐5.33)
Number of CS median (range)	Pre	1.00 (.00‐7.00)	3.00 (.00‐6.00)	1.00 (.00–6.00)
	Post	1.00 (.00‐3.00)	1.00 (.00‐6.00)	0.00 (.00‐6.00)

Abbreviations: CS, cluster seizures; MSDF, monthly seizure day frequency; MSF, monthly seizure frequency; rTMS, repetitive transcranial magnetic stimulation.

### 3.2 Trial II

Dogs previously treated by sham stimulation (n = 5) received active treatment. Median TMS coil output was 75% (range, 70‐90%). No dogs were lost to follow‐up. Significant differences were found in MSF (*P* = .03) and MSDF (*P* = .008) but not in number of CS (*P* = .12) post‐rTMS compared to pre‐rTMS. No changes in AED treatment were made during the evaluation period. The results are summarized in Table 2. Based on the MSF ratio, the median rTMS effect lasted for 4 months (range, 2‐6). Median follow‐up of the dogs from inclusion in the active treatment group until termination of the study was 5 months (range, 3‐6). No adverse effects related to the treatment were reported.

## 4 DISCUSSION

We investigated a new noninvasive and safe neurostimulation technique as a potential treatment option, adjunctive to AEDs, for dogs with IE and obtained preliminary results from application of 5‐day low‐frequency rTMS on small number of dogs with drug‐resistant IE. Our results provide an indication of the effect that can be expected and, as such, provide an ideal starting point to perform power calculations and design future, large‐scale studies to further assess the role of this neurostimulation method in veterinary medicine.

The principle behind rTMS is Faraday's law of electromagnetic induction.^25^, ^26^, ^27^ More precisely, short alternating electrical currents pass through a stimulation coil, which generates a secondary alternating magnetic field parallel and in the opposite direction to the primary electrical current. This field, when perpendicularly orientated to the head, can bypass the scalp and reach the cerebrospinal fluid and brain, unhindered by the skull and soft tissues. The magnetic field induces a secondary electrical current, which modulates the cortical neurons and produces the desirable neurobiological effects.^28^

The effects of rTMS are dependent on the frequency and pattern of the stimuli. Alterations in the stimulation parameters (ie, number of trains, number of pulses per train, TMS frequency, and intensity and duration of treatment) might affect specific neuronal cells, which could lead to selective cortical modulation adjusted for the specific disorder targeted.^29^ However, no consensus currently exists on the optimal stimulation parameters for specific diseases, such as epilepsy.^10^ Cortical excitability can be increased or decreased using high‐frequency (>1 Hz) and low‐frequency (≤1 Hz) neurostimulation, respectively, which likely is caused by the potential induction of long‐term potentiation and depressive mechanisms, respectively.^30^ Although there is an overall agreement that low‐frequency rTMS supresses epileptic discharges and leads to a decrease in seizure frequency,^31^, ^32^, ^33^ the remaining stimulation parameters are quite variable in trials of human patients.^10^ With regard to the pulses, more pulses per rTMS session were associated with higher efficacy.^34^ As far as the duration of treatment is concerned, rTMS has an effect that outlasts the duration of the treatment, which can be attributed to consecutive sessions.^14^, ^35^, ^36^ In 1 study, neuroplastic long‐lasting changes were observed when at least 2 rTMS sessions were administered within 24 hours, but not when administered 1 week apart.^35^ In addition, the effects of low‐frequency rTMS on cortical excitability were dependent on the intensity used.^37^ In 1 study, high rTMS intensity (90%) was found to be superior and significantly decreased seizure frequency, compared to low‐intensity rTMS (20%),^38^ whereas another study found good antiepileptic rTMS effect with relatively high intensity (70%).^14^ In our study, 1‐week (5 consecutive days), low‐frequency (1 Hz), high‐intensity (≥70%) rTMS with a high total number of pulses (1620 pulses) showed potentially promising results with regard to the efficacy and safety of this technique in dogs. The stimulation parameters used significantly decreased in MSF and MSDF, but not the number of CS post‐rTMS compared to pre‐rTMS, within the active rTMS group, which implied that the overall number and days of epileptic seizure events were decreased, but the number of CS events was not significantly affected.

Finally, different coil types can achieve different effects with respect to depth and focal distribution.^39^ The 8‐shaped coil provides more focal whereas the round coil provides more widespread currents on the cortical surface.^10^ Also, vertex stimulation might lead to downregulation of excitability within the entire epileptic network, and, thus, it is likely to provide good outcome in patients with generalized epilepsy.^34^, ^40^, ^41^, ^42^ In our study, because the epileptogenic zone responsible for generating the epileptic seizures was not identified and our patients suffered from generalized IE rather than a focal epileptogenic lesion, a round coil was used over the vertex to globally stimulate the cortex.

Although our results highlighted the promising potential and importance of investigating this new noninvasive neurostimulation technique as a treatment option for dogs with drug‐resistant IE, our study had some limitations that preclude definitive conclusions. Specifically, the low number of included subjects does not allow strong evidence‐based conclusions about the technique's efficacy. In addition, the results derived from trial II, although positive, have high risk of bias because trial II was an open‐label nonrandomized uncontrolled clinical trial. However, our study provides new insight into the nonpharmacological treatment of drug‐resistant IE in dogs and encourages further evaluation of rTMS in future large‐scale veterinary studies.

## 5 CONCLUSION

We presented preliminary evidence on the potential antiepileptic effect of rTMS in epileptic dogs that received active stimulation because such an effect was not shown in dogs that received sham rTMS, although the small study population did not allow sufficiently powered results to detect a statistical difference between the groups. Evidence also was provided to support the safety profile of this technique in dogs. Because the stimulation parameters used are critical for the extent and duration of antiepileptic effect, altering and optimizing the stimulation protocols on an individual basis might lead to longer lasting effects. Large‐scale trials in epileptic dogs evaluating this noninvasive neurostimulation technique and optimizing the stimulation protocols should be performed to substantiate our results and provide definitive conclusions with respect to the efficacy or rTMS in dogs with drug‐resistant IE.

## CONFLICT OF INTEREST DECLARATION

Authors declare no conflict of interest.

## OFF‐LABEL ANTIMICROBIAL DECLARATION

Authors declare no off‐label use of antimicrobials.

## INSTITUTIONAL ANIMAL CARE AND USE COMMITTEE (IACUC) OR OTHER APPROVAL

Approved by Ghent University ethical committee, approval number EC 2016‐130.

## HUMAN ETHICS APPROVAL DECLARATION

Authors declare human ethics approval was not needed for this study.
